# Dataset of wind blow sand erosion test on ultrasonic surface treated cementitious composites

**DOI:** 10.1016/j.dib.2020.105943

**Published:** 2020-06-27

**Authors:** Y Shi, ZM Shi

**Affiliations:** a School of Materials Science and Engineering, Inner Mongolia University of Technology, Hohhot, Inner Mongolia, PR China; b Inner Mongolia Autonomous Region Engineering Research Center of Structure Inspection, Appraisal and Safety Assessment, Inner Mongolia University of Technology, Hohhot 010051, PR China

**Keywords:** Ultrasonic, Surface treatment, Wind blown sand, Erosion, Cementitious

## Abstract

In this paper, we take cement mortar and paste as specimens, a novelty method named ultrasonic surface treatment(UST) was employed to form a hardening surface layer on cementitious specimens to improve its wind-blown sand erosion resistance, surface hardness and apparent density. The specimens with curing ages of 1-day, 3-days, 7-days, and 28-days were adopted. The wind blown sand erosion test was carried out in a wind-blown sand erosion test system, which simulated a wind blown sand environment of a wind speed of 30 m/s and a sand feed rate of 30 g/min. The erosion angle of 30°, 60°, 90° were adopted. The mass loss in erosion process was measured, then the erosion resistance was calculated. The surface hardness was tested with a Vickers micro hardness tester. The apparent density of cement paste was measured with mass volume method. The data provided reveal the improvement on wind blown sand erosion resistance, surface hardnenss and apparent density of cementitious materials with ultrasonic surface treatment. That may be used in the investigation on improving the erosion resistance and to evaluate the effectiveness of the UST method on cementitious materials.

**Specifications Table**

**Table:** 

**Subject**	Mechanics of Materials
**Specific subject area**	Erosion resistance of ultrasonic surface treated cementitious composites
**Type of data**	Tables
**How data were acquired**	The erosion resistance R_E_ Wind blown sand erosion tests were tested in a wind-blown sand erosion test system, as shown in Fig. 1. The erosion resistance *R_E_*(in cm^3^/cm^3^) that defined as the ratio between volumetric solid particle consumption and removed target material volume, was calculated in accordance with Eq. (1) . The surface hardness was tested with a Vickers micro hardness tester, the surface of the UST and the contrast mortar specimens were polished properly before test. The apparent density was measured with mass volume method.
**Data format**	Analyzed
**Parameters for data collection**	With a wind-blown sand erosion test system, the wind speed was 30 m/s and sand feed rate was 30 g/min; the diameter of the nozzle was 10 mm, the space between the specimens and the nozzle was 100 mm. Erosion angles were 30°, 60°, and 90°. The specimens with curing ages of 1-day, 3-days, 7-days, and 28-days were adopted. The surface hardness was tested with force of 0.1 kgf and a loading time of 10 s for each test point.
**Description of data collection**	The total erosion duration was 30 min, and within 10 min the mass loss was measured each minute. Then, the mass loss was measured every 5 min. 9 specimens in a group for each curing age and 3 specimens in a group for each test angle. The contrast specimens had the same quantity. Then the erosion resistance *R_E_* was calculated in according to Eq. (1). The surface hardness test, 33 data points were collected on each specimens and a 2 mm space between each sampling point was adopted. 3 specimens in a group. The apparent density of cement paste specimens was measured with growth of curing age, dried before testing, 3 specimens in a group.
**Data source location**	Inner Mongolia University of Technology Hohhot, Inner Mongolia, China North latitude 40.846° and east longitude 111.677°.
**Data accessibility**	With the article
**Related research article**	Shi Y, Shi ZM. Ultrasonic surface treatment for improving wind-blown sand erosion resistance of cementitious materials, WEAR

**Value of the Data**

The data provided in this article revealed the improvement on wind blow sand erosion resistance, surface hardness and the apparent density of cementitious materials that treated with ultrasonic surface treat(UST) method.

These data can be used in the investigation on improving the erosion resistance and to evaluate the effectiveness of the UST method on cementitious materials.

## 1 Data description

Tables 1–4 is the erosion resistance *R_E_* of 1, 3, 7, and 28day age cement mortar specimens. Table 5 is the accumulate erosion resistance *R_E_* in a 30 min erosion.

**Table 1:** Erosion resistance of 1day age specimens(cm^3^/cm^3^).

Erosion time(minutes)		1	2	3	4	5	6	7	8	9	10	15	20	25	30
30°	CS	621	706	1312	1640	2187	2417	2701	2870	3532	3280	3764	4415	4685	5339
	CS	429	883	1391	1996	2296	1837	2296	2701	2551	2551	2733	3532	3891	3891
	CS	516	778	1312	1583	2417	2187	2551	2551	2701	3061	3427	3644	4028	4783
	UV	1177	2417	2551	2701	3280	3061	3061	3826	3532	4592	4885	6377	6957	7653
	UV	717	1583	1481	2087	2417	2551	2870	2870	3280	3061	3587	4332	4685	5600
	UV	753	1766	2087	2417	2551	2870	3061	3061	3826	4174	4502	4991	5887	6560
60°	CS	510	937	1068	1148	1241	883	1148	1701	1531	1701	2032	1979	1913	1929
	CS	425	675	778	957	1093	957	1148	1093	1068	1481	1444	1664	1628	1435
	CS	483	998	1068	1391	1391	1312	1837	1701	1837	1837	1882	2087	2087	2106
	UV	820	1241	1531	1913	1766	1913	1996	2296	1913	2296	2982	3189	3376	3587
	UV	638	1020	1391	1312	1481	1766	1837	1996	2187	1837	2733	2982	3061	3376
	UV	685	1481	2187	2087	2087	2087	2087	2087	2296	2701	3189	3234	3376	3427
90°	CS	665	1120	1275	1177	1435	1481	1531	1766	1837	1640	1822	2087	2417	2319
	CS	638	1275	1435	1351	1531	1701	1701	2087	1996	2417	2523	2319	2800	2391
	CS	560	741	820	918	1068	1093	1044	1177	1177	1068	1290	1367	1501	1851
	UV	835	1435	1583	1766	1996	1837	1837	3061	2701	2701	3061	3427	3427	3644
	UV	883	1531	1701	1766	1701	1996	1913	2417	2551	2551	3376	3479	4174	3826
	UV	850	464	1275	1435	1312	1913	1996	1837	1837	2087	2367	2701	2701	2251

**Table 2:** Erosion resistance of 3day age specimens(cm^3^/cm^3^).

erosion time(minutes)		1	2	3	4	5	6	7	8	9	10	15	20	25	30
30°	CS	1351	3061	3532	3826	4174	2551	3826	5740	5102	3532	5600	7174	7917	8830
	CS	866	2087	2187	3280	3532	3532	3532	3826	3826	3826	4174	5339	5740	6560
	CS	1093	1583	2087	3280	3532	3532	4174	4592	4174	4592	4592	7406	9566	9982
	UV	1093	2187	2551	3826	2870	2417	3826	4592	6560	5102	5466	8830	10,436	11,479
	UV	1391	2701	3280	4174	4174	3826	4174	4592	5102	5102	4991	6560	8503	9982
	UV	977	2296	2551	3280	2870	4174	4174	4174	2701	5740	6205	7917	9566	10,436
60°	CS	1260	1620	2001	1790	2087	2551	2616	2268	2126	2429	2538	3092	3401	3209
	CS	709	1260	1479	1583	1620	1790	1890	1790	2126	2126	2501	2609	2616	2882
	CS	945	1479	1790	2126	2296	2429	2616	2834	2834	2834	3209	3037	3543	3779
	UV	972	1701	2268	2268	2551	3401	2616	3092	2429	3092	3587	3958	4361	4596
	UV	1000	1620	1790	2001	2429	2616	2429	2616	2834	2616	3270	3955	3779	4148
	UV	773	1361	2001	1890	2429	2001	2616	2429	2834	2834	3270	3543	3779	3618
90°	CS	1068	1701	2296	2296	1996	2187	2087	2701	2187	2701	3145	3532	3703	3532
	CS	1044	1837	2087	2087	2087	2417	2551	2551	2551	2551	2982	3764	3891	4100
	CS	866	1275	1640	1701	2087	1913	2087	2187	2187	2296	2906	3479	3587	4174
	UV	866	1640	2087	2551	2870	2296	2870	2701	2870	3061	3826	4174	4415	4685
	UV	1148	1640	1837	2296	1996	2187	2701	2701	2870	3061	3280	3826	4415	4252
	UV	806	1391	1766	1837	2087	1996	2296	2296	2417	2551	3102	3764	4502	4991

**Table 3:** Erosion resistance of 7day age specimens(cm^3^/cm^3^).

Erosion time(minutes)		1	2	3	4	5	6	7	8	9	10	15	20	25	30
30°	CS	1338	2453	2759	2870	2870	3061	2943	2943	3679	4014	6132	6132	7884	8830
	CS	1226	2324	3280	3154	3679	3154	3154	3061	6307	4014	6132	7358	8176	8830
	CS	849	1766	2597	3679	3154	3532	3826	3679	4906	6307	6493	7612	8176	11,038
	UV	1208	2087	2870	3826	5102	4592	5102	4592	4592	4592	6560	7917	8830	8503
	UV	1312	1996	2701	3826	3826	3826	4174	3826	4592	5102	6042	7406	8830	9982
	UV	806	2296	3280	3061	3532	5102	3826	5740	4592	5102	6957	8199	8503	10,436
60°	CS	900	1913	2187	2296	2187	2551	2417	2087	2417	2187	3327	3532	3958	3958
	CS	866	1640	1701	1913	2087	2187	2296	2187	2296	2551	3532	3764	4252	4592
	CS	765	1481	1701	1996	2087	2087	2296	2187	2296	2296	3102	3479	3703	4028
	UV	977	1766	1996	2296	2296	2417	2551	3061	3061	2870	3532	4028	4252	4685
	UV	883	1837	2087	2296	2187	2296	2551	2701	2870	3061	3587	4174	4502	4885
	UV	850	1640	1837	1913	2087	1996	2296	2296	2296	2551	3427	3764	4332	4592
90°	CS	1093	1701	1837	1996	1913	2087	2087	2417	2187	2296	2495	2943	3021	3532
	CS	957	1640	1837	1837	2087	2296	2296	2417	2701	2417	3532	3644	3764	4252
	CS	1068	1913	2187	2296	2187	2417	2551	2551	2870	3061	3764	4100	4592	4991
	UV	883	1701	1837	2417	2296	2701	2701	3532	3061	3280	3427	3958	4415	5102
	UV	900	1913	2187	2296	2296	2870	3061	3061	3826	3532	4100	4783	5600	6377
	UV	850	1640	1766	1996	2417	2087	2187	2296	2701	2870	3587	3891	4332	5466

**Table 4:** Erosion resistance of 28day age specimens(cm^3^/cm^3^).

Erosion time(minutes)		1	2	3	4	5	6	7	8	9	10	15	20	25	30
30°	CS	1391	1996	2551	2417	2701	4174	4592	5102	4592	5102	6752	8503	10,436	6752
	CS	1208	1640	2087	2701	3061	3280	3532	5740	5102	3826	5466	7174	8199	9183
	CS	1208	1766	2187	2417	3061	3826	4174	4592	5102	5102	5600	7917	8503	9982
	UV	1837	3280	3826	5740	5102	7653	5740	6560	6560	9183	8503	8503	11,479	9982
	UV	1435	2701	3280	3826	3826	4174	4174	4174	5740	4592	5339	6377	7174	8199
	UV	1391	2551	3280	3532	4174	4174	5102	5740	6560	6560	7174	8199	9183	9982
60°	CS	1068	1701	2296	2296	2417	2417	2417	2417	2551	3061	3061	4332	3764	3327
	CS	937	1701	1837	2087	1913	2296	2417	1996	2551	2701	3327	3587	4252	3826
	CS	957	1766	1913	2296	2417	2551	2296	2187	2417	2701	3102	3532	3958	4252
	UV	1351	1351	2087	2870	2417	3280	2870	2870	3061	3532	3958	4332	4685	4885
	UV	883	1391	1766	2087	2087	2087	2087	2296	2417	2551	3764	3891	4028	3826
	UV	1177	1701	1996	2296	2417	2551	2870	3061	3061	2870	4332	4685	5218	5102
90°	CS	1208	2087	2296	2417	2417	3280	2417	2701	2701	3061	3427	4174	3958	4028
	CS	1312	1996	2187	2551	2187	2870	2870	2870	3061	2870	3703	3891	4592	4783
	CS	1020	1766	2187	2551	3061	2417	2870	3280	3061	3061	4028	4502	4685	4332
	UV	1435	2187	2870	2701	3061	3280	3826	3532	4174	3280	4991	4885	6752	7174
	UV	1093	1583	1913	2417	2551	2701	3061	3061	2701	3280	3427	3891	4685	5740
	UV	1701	2417	2551	3280	2870	3061	3532	3061	3532	3826	4415	4991	4885	7406

**Table 5:** Accumulate erosion resistance R_E_(cm^3^/cm^3^) in 30 min.

Curing time	1d curing		3d curing		7d curing		28d curing		Erosion angle
Species	CS	UV	CS	UV	CS	UV	CS	UV	
1	2717	4332	5046	4991	4564	5238	4991	6957	30°
2	2296	3054	3848	5140	4854	5083	4546	4973	
3	2482	3569	4546	4833	4696	4973	4717	5764	
1	1222	2438	2573	3152	2834	3102	2888	3393	60°
2	1644	2152	2101	2907	2846	3109	2805	2794	
3	1441	2532	2730	2699	2624	2840	2852	3452	
1	1750	2570	2777	3152	2478	3124	3152	4252	90°
2	1996	2685	2876	3008	2834	3550	3311	3241	
3	1227	1851	2560	2783	3218	2969	3327	4075	

In above table, *R_E_*(in cm^3^/cm^3^) were calculated in accordance with Eq. (1).

(1) $$R_{E}=\frac{\rho_{M}m_{p}}{\Delta m\rho_{p}}$$

In Eq. (1), *ρ_M_* is the target material density, that is 2.204 g/cm^3^; *m_P_* is the mass of grit consumption, that is 30 g/min; *ρ_P_* is the grit bulk density, that is 1.440 g/cm^3^; ∆m is the target specimen mass loss. The total erosion duration was 30 min, and within 10 min the mass loss was measured each minute. Then, the mass loss was measured every five minutes.

Table 6 is the surface hardness of the specimens with curing age of 1, 3, 7, 14 and 28 days.

**Table 6:** Micro-hardness of cement mortar(MPa) (HV/0.1/10).

Curing time	1d curing		3d curing		7d curing		14d curing		28d curing	
Species	CS	UV	CS	UV	CS	UV	CS	UV	CS	UV
	9	33	36	28	12	88	32	71	37	69
	10	53	23	22	23	55	29	64	73	112
	13	31	14	17	17	59	21	53	19	121
	13	36	12	22	20	86	26	86	30	98
	12	13	15	33	24	65	30	80	23	70
	14	36	21	37	21	49	22	88	41	98
	15	51	18	26	13	71	25	88	35	102
	10	33	20	32	15	92	25	79	48	103
	9	13	21	50	17	74	23	80	56	110
	15	33	15	46	26	82	24	66	43	117
	12	42	28	41	21	74	24	71	50	127
	10	24	13	26	13	69	30	58	48	143
	10	46	15	65	16	56	23	48	42	138
	12	26	17	54	18	39	14	80	33	147
	13	21	20	35	20	76	35	30	32	99
	12	27	19	71	22	108	33	106	66	119
	12	35	17	59	19	52	23	71	36	98
	13	47	18	60	20	56	15	76	41	137
	20	35	17	55	15	52	31	52	52	102
	13	37	15	60	17	88	28	47	49	120
	17	37	18	92	17	74	23	61	40	94
	18	34	16	71	22	40	31	103	52	81
	24	30	8	74	16	54	20	143	50	90
	19	23	11	52	10	52	27	122	53	123
	16	37	12	42	22	61	23	80	52	96
	18	44	18	76	17	77	33	105	73	110
	13	33	18	71	17	82	28	96	63	94
	14	27	15	74	20	80	23	57	59	135
	13	29	15	110	32	73	24	69	37	118
	13	31	17	88	18	75	32	92	50	102

Table 7 is the apparent density of the cement paste specimens that cured for 1, 3, 7, 14, 21 and 28 days.

**Table 7:** Apparent density of the CS and UV cement paste(g/cm^3^).

Curing time	1d	3d	7d	14d	21d	28d
CS	1.941	1.982	2.012	2.034	2.037	2.051
	1.914	1.957	1.995	2.028	2.036	2.057
	1.933	1.958	2.001	2.026	2.029	2.035
UV	2.016	2.032	2.060	2.083	2.095	2.119
	2.003	2.031	2.057	2.076	2.087	2.106
	2.025	2.043	2.068	2.088	2.102	2.121

**Fig. 1:**
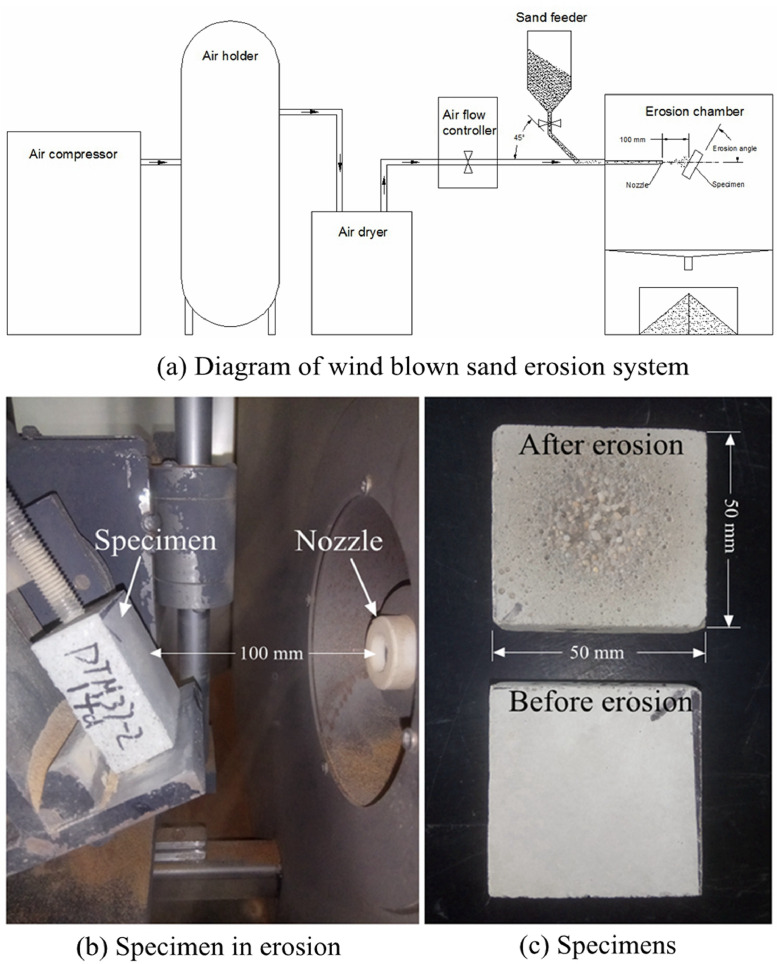
Erosion test equipment and specimens.

In these tables, “UV” represents specimens which were treated with ultrasonic, “CS “ represents the contrast specimens (Fig. 1).

## 2 Experimental design, materials, and methods

### 2.1 Experimental design

Wind blown sand erosion affecting the durability of cementitious composites in a windy sand environment [1,2]. Most researches focused on the erosion mechanism [3], [4], [5], a few studies focused on improving the erosion resistance [6,7]. In this paper, we take cement mortar and paste as specimens, a novelty method named ultrasonic surface treatment(UST) was employed to form a hardening surface layer on specimens to improve its wind-blown sand erosion resistance and surface hardness, and to improve the apparent density of the cement paste.

Cement mortar specimens treated with and with out UST method were prepared for wind blown sand erosion test and surface hardness test, 9 specimens in a group for each curing age and 3 specimens in a group for each erosion angle, and the contrast specimens had the same quantity. Cement paste specimens treated with and with out UST method were prepared for apparent density measurement.

### 2.2 Materials

The specification of cement was PⅡ52.2, the aggregate was ISO standard sand, The erosion particles was aellian sand collected in Hobq Desert located at the Ordos Plateau, Inner Mongolia, China.

### 2.3 Methods

**Fig. 2:**
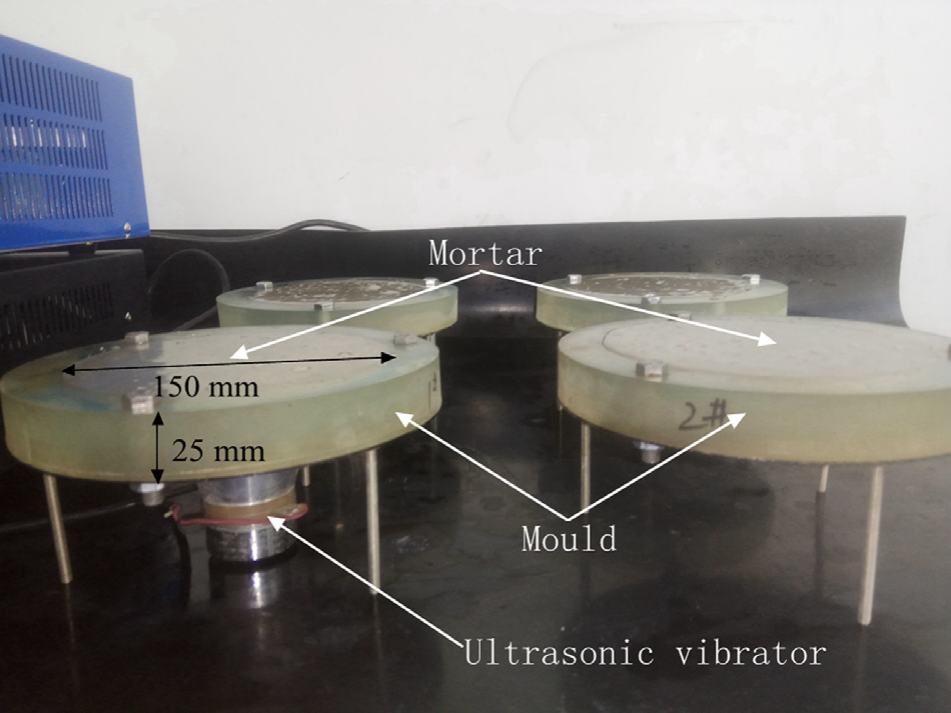
UST moulds and specimens production.

The composition and the mixing of the mortar specimens was in accordance with ISO 679:2009(E) [8]. The ultrasonic surface treatment was applied on mortar surface 30 min after pouring with a special mould, as shown in Fig. 2. The ultrasonic vibration power was 30 W with a duration of 30 min. The specimens were cut into blocks with a length of 50 mm and a thickness of 25 mm with curing age of 1, 3, 7, 28 days. The wind blown sand test was carried out in a wind-blown sand erosion test system, the wind speed was 30 m/s and the sand feed rate was 30 g/min, the erosion angle was 30, 60, 90°. The mass was weighed and the erosion resistance was calculated.

The surface hardness was tested with a Vickers micro hardness tester, the surface of the UST and the contrast specimens were polished properly. The data of the specimens with curing age of 1, 3, 7, 14 and 28 day were collected. 33 data points on each specimens and 2 mm space between each sampling point was adopted.

The apparent density was tested in accordance with mass-volume method. The cement paste specimens were cut into rectangles and the volume was measured, the mass was weighed with curing age of 1, 3, 7, 14, 21, 28 days, then the apparent density was calculated. The specimens were dried in 60 °C for 3 h before weighing.

## Declaration of Competing Interest

The authors declare that they have no known competing financial interests or personal relationships which have, or could be perceived to have, influenced the work reported in this article.

## Appendix Supplementary materials

Image, application 1
